# An Induced Mutation in Tomato eIF4E Leads to Immunity to Two Potyviruses

**DOI:** 10.1371/journal.pone.0011313

**Published:** 2010-06-25

**Authors:** Florence Piron, Maryse Nicolaï, Silvia Minoïa, Elodie Piednoir, André Moretti, Aurélie Salgues, Dani Zamir, Carole Caranta, Abdelhafid Bendahmane

**Affiliations:** 1 Unité de Recherche en Génomique Végétale, UMR INRA-CNRS-Uni. EVRY, Evry, France; 2 Unité de Génétique et Amélioration des Fruits et Légumes, INRA, UR1052, Montfavet, France; 3 Institute of Plant Sciences, Faculty of Agriculture, The Hebrew University of Jerusalem, Rehovot, Israel; University Medical Center Groningen, Netherlands

## Abstract

**Background:**

The characterization of natural recessive resistance genes and Arabidopsis virus-resistant mutants have implicated translation initiation factors of the eIF4E and eIF4G families as susceptibility factors required for virus infection and resistance function.

**Methodology/Principal Findings:**

To investigate further the role of translation initiation factors in virus resistance we set up a TILLING platform in tomato, cloned genes encoding for translation initiation factors eIF4E and eIF4G and screened for induced mutations that lead to virus resistance. A splicing mutant of the eukaryotic translation initiation factor, S.l_eIF4E1 G1485A, was identified and characterized with respect to cap binding activity and resistance spectrum. Molecular analysis of the transcript of the mutant form showed that both the second and the third exons were miss-spliced, leading to a truncated mRNA. The resulting truncated eIF4E1 protein is also impaired in cap-binding activity. The mutant line had no growth defect, likely because of functional redundancy with others eIF4E isoforms. When infected with different potyviruses, the mutant line was immune to two strains of *Potato virus Y* and *Pepper mottle virus* and susceptible to *Tobacco each virus*.

**Conclusions/Significance:**

Mutation analysis of translation initiation factors shows that translation initiation factors of the eIF4E family are determinants of plant susceptibility to RNA viruses and viruses have adopted strategies to use different isoforms. This work also demonstrates the effectiveness of TILLING as a reverse genetics tool to improve crop species. We have also developed a complete tool that can be used for both forward and reverse genetics in tomato, for both basic science and crop improvement. By opening it to the community, we hope to fulfill the expectations of both crop breeders and scientists who are using tomato as their model of study.

## Introduction

Tomato (*Solanum lycopersicum* L.) belongs to the Solanaceae family that contains about 2800 species and many agriculturally valuable crops. For decades, tomato has played key roles in the field of plant molecular biology, serving as an excellent model organism for investigating plant–pathogen interactions [1], fruit development [2], ripening processes [3], [4], [5], [6], sugar metabolism [7], [8], [9], carotenoid biosynthesis [10], [11], quantitative trait locus (QTL) analysis [12], and plant architecture [13].

The genome structures of most of the solanaceous plants are relatively well conserved [14]. Tomato is the most intensively researched Solanaceae with the availability of extensive genetic and genomics resources including interspecific introgression lines collection, large collections of wild relatives and mutants with characterized phenotypes, microarrays with approximately 12 000 unigenes designed based on large collections of ESTs [15], [16], and metabolome database of tomato fruit [17]. With the completion of the genome sequencing project in the near future [18], a major challenge is to determine gene functions. In plants, the most common techniques to produce altered or loss of function mutations are T-DNA or transposon insertional mutagenesis [19] and RNA interference [20]. However, unless a high-throughput transformation protocol becomes available for tomato, functional analysis of tomato genes with the tagging approaches is not realistic. On the other hand, ethyl methanesulfonate (EMS) mutagenesis is a straightforward and cost-effective way to saturate a genome with mutations [21]. TILLING (Targeting Induced Local Lesions IN Genomes) uses EMS mutagenesis coupled with gene-specific detection of single-nucleotide mutations [22], [23], [24]. This strategy generates allelic series of the targeted genes which makes it possible to dissect the function of the protein as well as to investigate the role of essential genes that are otherwise not likely to be recovered in genetic screens based on insertional mutagenesis. This reverse genetic strategy encompasses all types of organisms and can be automated in a high throughput mode [25], [26], [27], [28].

To investigate the capacity of TILLING as a powerful tool of reverse genetics in tomato and to identify novel alleles of agronomic importance, we have set up a tomato TILLING platform and performed a screen for mutations in host factors required for the potyvirus infection. The genus Potyvirus is the largest among plant viruses and includes the widespread and destructive viruses for a number of crops worldwide. The potyviral genome consists of a single-stranded, positive-sense RNA molecule that contains at the 5′-end a covalently linked virus-encoded protein named VPg, replacing the cap structure of mRNA and required for viral infection [29], [30]. In recent years, the molecular cloning of recessive resistance genes to RNA viruses led to the identification of a new class of resistance genes corresponding to mutations in translation initiation factors, including the eukaryotic initiation factor 4E (eIF4E) [31], [32] and to a lesser extent, the eukaryotic initiation factor 4G (eIF4G) [33]. The majority of eIF4E-mediated Potyvirus resistances are mediated by a small number of amino acid changes in the eIF4E protein [31], [32]. The exact mechanism by which eIF4E mutations control resistance is still unclear but several results argue in favor of an altered function induced by these amino acid mutations with respect to VPg binding [34], [35], [36]. eIF4E like other factors from the translation initiation complexes belongs to a small multigenic family encoding for two protein isoforms, eIF4E and eIF(iso)4E [37]. Interestingly, complete resistance to Potyvirus may result from mutations in a single *eIF4E* or from combined mutations in different paralogs, depending on the virus ability to use one or several eIF4E to perform its infectious cycle [38], [39].

In tomato, the role of eIF4E in resistance to two potyviruses, *Potato virus Y* (PVY) and *Tobacco etch virus* (TEV), was demonstrated by the molecular cloning of the recessive resistance gene *pot-1*. *pot-1* encodes for the eIF4E1 protein and the resistant and susceptibility alleles differ by 4 amino acid substitutions [40], [41]. To investigate further the role of translation initiation factors eIF4E and eIF4G in virus resistance, we first set up a tomato TILLING platform, exploiting the M82 EMS-mutageneized population described previously ([42] http://zamir.sgn.cornell.edu/mutants/). Then, we screened for mutations in the five translation initiation factors, eIF4E1, eIF4E2, eIF(iso)4E, eIF4G and eIF(iso)4G identified in tomato using the TILLING approach. The mutant lines were characterized with respect to potyvirus resistance and translation of mRNA with the objectives to get insights into molecular mechanisms underlying translation initiation factors-mediated resistance to potyviruses. In this analysis, a splicing mutant of eIF4E1 was found immune to two strains of *Potato virus Y* and *Pepper mottle virus* and susceptible to *Tobacco each virus*.

## Results

### Set up of the M82 TILLING platform

To set up the TILLING platform we exploited the tomato M82 EMS-mutagenized population described previously [42]. M82 is an inbred variety with determinate flowering that perform well under various growth environments and is amenable to fast screens in seedling trays and pots as well as in field conditions. M82 mutant population was visually phenotyped in the field and categorized into a morphological catalogue that can be searched and accessed via the web (http://zamir.sgn.cornell.edu/mutants/). DNA samples were prepared from 4759 M3 families, each representing an independent M1 family and organized in pools of 8 families. One key factor in TILLING is the availability of the annotated genomic sequence of the gene to be tilled, which in this case was facilitated by the sequencing of the tomato euchromatin genomic region and the availability of high number of ESTs. The CODDLE software (Codons Optimized to Discover Deleterious Lesions) [43] combined with the PRIMER3 tool [44] were used to define the best amplicon for TILLING. Mutations were detected in the amplified targets using the mismatch-specific endonuclease ENDO1, as described previously [45].

A primary objective in TILLING is to exploit a mutant population where every locus is mutated and represented by multiple alleles. To evaluate the existence of multiple alleles per locus, we calculated the mutation frequency in 19 targeted genes (Table 1) according to [21]. Mutation frequency equals the size of the amplicon multiplied by the total number of samples screened and divided by the total number of identified mutants. We estimated the average mutation rate to one mutation every 574 kb (Table 1). In the 19 selected genes, we identified 256 nucleotides changes among which 145 were exonic mutations (Table 1). We obtained from 2 to 43 alleles for each target. Induced mutations discovered in exons consisted of 58.6% missense, 36.6% silent and 4.8% translation stop or splice junction mutations (Table 2). The number of silent mutations was higher than the CODDLE-predicted proportion. In contrast missense, stop and splice junction mutations were recovered in a slightly lower proportion than predicted (Table 2).

**Table 1:** Tilled genes and mutation density in the M82 mutant population.

Gene Name	Amplicon size (bp)	Identified mutants	Missense mutation	Null mutation	Silent mutation	Intronic mutation	Mutation density
eIF4E1	1 633	7	1	1^*^	1	4	1/1110 kb
eIF4E2	324	3	0	1	0	2	1/514 kb
eIF(iso)4E	1 340	14	3	1	2	8	1/455 kb
eIF4G	3 609	43	20	0	16	7	1/400 kb
eIF(iso)4G	2 017	16	4	0	6	6	1/600 kb
DET1	2 646	23	7	0	4	12	1/547 kb
COP1like	979	4	1	3	0	0	1/1165 kb
DDB1a	1 216	11	3	0	5	3	1/526 kb
COP10	2 213	32	1	0	3	28	1/329 kb
NAM	1 638	5	4	0	1	0	1/1559 kb
ACO1	1 784	8	4	0	2	2	1/1061 kb
E8	1 810	6	2	0	3	1	1/1436 kb
DHS	638	2	1	0	0	1	1/1518 kb
RAB11a	407	2	2	0	0	0	1/968 kb
PG	1 943	28	4	0	0	24	1/330 kb
MET1	4 015	32	24	0	7	1	1/597 kb
Exp1	1 025	8	1	1	0	6	1/610 kb
CRTISO	1 011	9	2	0	1	6	1/535 kb
CUL4	629	3	1	0	2	0	1/998 kb
**Total/Mean**	**30 877**	**256**	**85**	**7**	**53**	**111**	**1/574 kb**

M82 mutant population was screened for mutations in the listed genes. The total size of the screened amplicons, for each gene, the number of mutants identified and the mutation frequency for each amplicon are indicated. The average mutation frequency was estimated to one mutation per 574 kb and is calculated as described previously [21], except that the sizes of all the amplicons were summed and divided by the total number of identified mutants.

**Table 2:** Comparison of expected and observed types of mutations in tilled exonic regions.

	All mutations	Silent changes	Missense changes	Nonsense changes	Splice junction changes
Distribution observed	145	53	85	6	1
Percent Observed	100	36.6	58.6	4.1	0.7
Percent Expected	100	28.1	65.2	5.5	1.2

The percentage of expected mutations was calculated based on the CODDLE analysis of the tilled exonic regions.

### EMS mutation screening in translation initiation factors 4E and 4G

**Figure 1:**
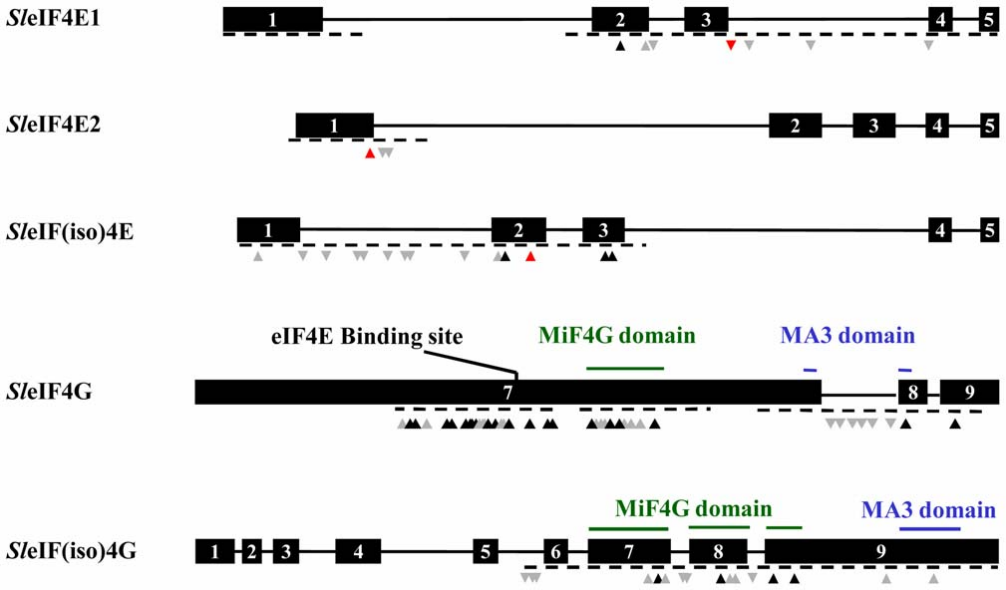
Representation of induced mutations in *Sl-eIF4E1*, *Sl-eIF4E2*, *Sl-eIF(iso)4E*, *Sl-eIF4G* and *Sl-eIF(iso)4G*. Black boxes represent the exons. Lanes linking exons indicate introns. Dashed lines indicate the genomic regions screened for mutations. Triangles pointing up indicate mutations in coding regions, whereas those pointing down indicate mutations in noncoding regions. Red, black and grey triangles represent alterations causing truncations, missense and silent mutations, respectively. Only exons 7 to 9 are shown for *Sl-eIF4G*.

The molecular cloning of recessive resistance genes in crop species demonstrated that amino acid changes in translation initiation factors led to resistance to specific RNA viruses, including potyviruses [31], [32]. To test whether new resistance alleles could be engineered by TILLING, the M82 mutant collection was screened for mutations in genes encoding for translation initiation factors eIF4E and eIF4G. Genomic sequences required for mutation screening were inferred from the following cDNAs or EST: Genbank accession AF259801 and TIGR tentative consensus accessions TC126316 and TC126421 for *eIF4E* genes and TIGR tentative consensus accessions TC167837, TC 156946, TC 165028 and TC155154 for *eIF4G* genes. Two homologs of *eIF4E* were identified that shares 74% sequence identity and are referred hereafter as *Sl-eIF4E1* and *Sl-eIF4E2*; the other genes are referred as *Sl-eIF(iso)4E*, *Sl*-*eIF4G* and *Sl*-*eIF(iso)4G* based on their conserved genes structures and sequence identities with *Arabidopsis thaliana* orthologs (Figure 1).

We screened for mutations in the whole exonic regions of *Sl*-*eIF4E1*, and focalized the screening for other genes only on regions where natural mutations were shown to lead to virus resistance *i.e.*, exons 1 to 3 for *Sl-eIF(iso)4E*, exon 1 for *Sl-eIF4E2* and MiF4G, MA3 and eIF4E-binding domains for *Sl-eIF4G* genes (Figure 1). TILLING of *Sl*-*eIF4E1* yielded seven independent point mutations, which correspond to one silent, four intronic, one missense and one splicing site mutations (Table 3). TILLING of *Sl*-*eIF4E2* yielded two intronic mutations and one stop codon (W85Stop) mutations (Figure 1). TILLING of *Sl*-*eIF(iso)4E* yielded fourteen independent point mutants, which correspond to two silent, eight intronic, three missense and one stop codon (W105Stop) mutations (Table 3). TILLING of *Sl*-*eIF4G* yielded fourty three point mutations, which correspond to sixteen silent, seven intronic and twenty missense mutations (Table 3). Finally, TILLING of *Sl*-*eIF(iso)4G* yielded sixteen point mutations, which correspond to six silent, six intronic and four missense mutations (Table 3).

**Table 3:** Mutations discovered in *Sl-eIF4E1*, *Sl-eIF4E2*, *Sl-eIF(iso)4E*, *Sl-eIF4G*, and *Sl-eIF(iso)4G*.

Gene name	GenBank Accession n°	Nucleotide changes	Amino acid changes
***Sl*** **-eIF4E1**	**GQ451830**	G1171A	D/N
		G1242A	L =
		G1485A	Splicing site
***Sl*** **-eIF4E2**	**GQ451831**	G254A	W/Stop
***Sl*** **-eIF(iso)4E**	**GQ451832**	G57A	E =
		C878T	S =
		G882A	V/I
		G967A	W/Stop
		G1213A	S/N
		G1225A	S/N
***Sl*** **-eIF4G**	**GQ451834**	A1602T	K/I
		G1614A	R/K
		C1860T	S/F
		C1899T	A/V
		C1996T	A =
		G2042A	D/N
		C2058T	T/I
		A2064T	N/I
		T2072A	L/I
		C2202T	T/I
		G2236A	E =
		G2239A	K =
		G2239A	K =
		G2246A	E/K
		G2366A	E/K
		C2406T	P/L
		C2411T	L =
		C2411T	L =
		G2618A	E/D
		C2635T	S =
		C2635T	S =
		G2637A	S/N
		A2659T	E =
		G3068A	D/N
		C3095T	L =
		C3136T	D =
		G3192A	G/D
		G3272A	A/T
		C3277T	A =
		C3290T	L =
		C3377T	E =
		C3377T	E =
		G3427A	E =
		A3689T	N/Y
		A5490T	S/C
		G5647A	V/I
***Sl*** **-eiF(iso)4G**	**GQ451835**	G2114A	R =
		G2157A	E/K
		G2174A	K =
		G2438A	M/I
		G2477A	Q =
		C2486T	P =
		C2682T	S/F
		C2771T	P/S
		C3182T	L =
		G3397A	R =

Only exonic mutations are shown. The position of the mutations are indicated relative to the first base of the GenBank sequences.

### The *Sl*-eIF4E1 G1485A splice junction mutant is resistant to several potyviruses

**Figure 2:**
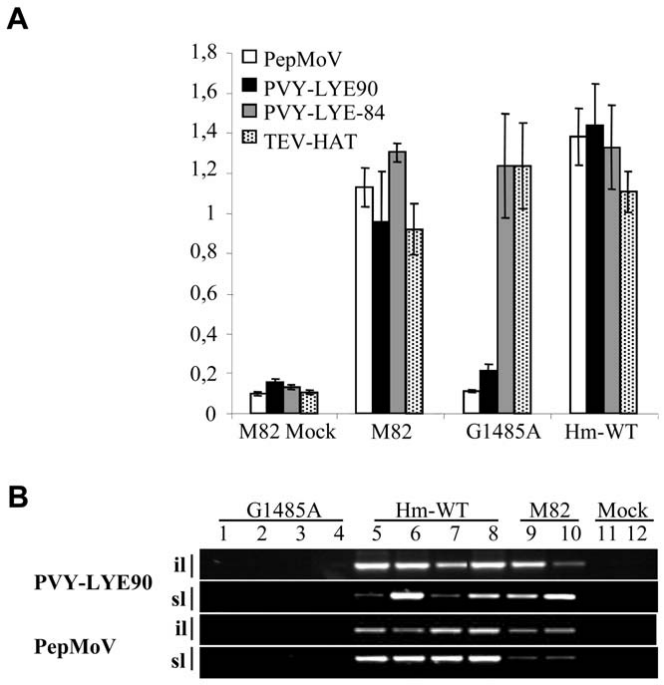
Potyvirus resistance assays of the *Sl*-eIF4E1 G1485A splicing mutant. *Sl*-eIF4E1 G1485A mutant and the corresponding Hm-WT were inoculated with PVY-LYE90, PVY-LYE84, TEV-HAT or PepMoV-Texas. (**A**) At 15 dpi, plants were assayed for potyviral coat protein accumulation by DAS-ELISA in non-inoculated leaves. (**B**) PVY-LYE90 and PepMoV RNA accumulation was assessed by RT-PCR in inoculated (il) and systemic leaves (sl). Mock indicates non inoculated M82 plants.

To test whether induced mutations in translation initiation factors could confer resistance to potyviruses, mutant lines affected in eIF4E or eIF4G proteins were challenged with three potyviruses infecting tomato, PVY (strains PVY-LYE90 and PVY-LYE84), TEV (strain TEV-HAT) and PepMoV (strain Texas). Lines derived from the same M3 family but homozygous wild type for the mutation, hereafter named Hm-WT, were used as control in the resistance assays, together with the susceptible cv. M82. All the controls and the TILLING mutant lines, except the *Sl*-eIF4E1 G1485A mutant, inoculated with PVY-LYE90, PVY-LYE84, TEV-HAT or PepMoV-Texas presented mosaic symptoms in apical non-inoculated leaves and exhibited high double antibody sandwich (DAS)-ELISA values at fifteen days post-inoculation (Figure 2A). The *Sl*-eIF4E1 G1485A mutant was immune to PVY-LYE90 and PepMoV-Texas infection. PVY-LYE90 and PepMoV-Texas RNAs were never detected in inoculated or non-inoculated leaves of the G1485A mutant line, indicating that viral accumulation is impaired at an early stage of the infection process (Figure 2B). *Sl-*eIF4E1 G1485A mutant line was, however, still susceptible to PVY-LYE84 and TEV infection (Figure 2).

To confirm this phenotype, *Sl*-eIF4E1 G1485A mutant line was backcrossed and the heterozygous line was self pollinated. Forty four plants obtained from the selfing of *Sl*-eIF4E1 G1485A heterozygous, consequently segregating for the mutation, were challenged with PVY-LYE90 and PepMoV and genotyped for the mutation. All Hm-WT and heterozygous plants were susceptible to PVY-LYE90 and PepMoV whereas all plants homozygous for the mutation were resistant to both viruses, confirming that resistance indeed results from mutation in *Sl-eIF4E1*.

### The *Sl*-eIF4E1 G1485A is a splicing mutant that encodes for a truncated mRNA

**Figure 3:**
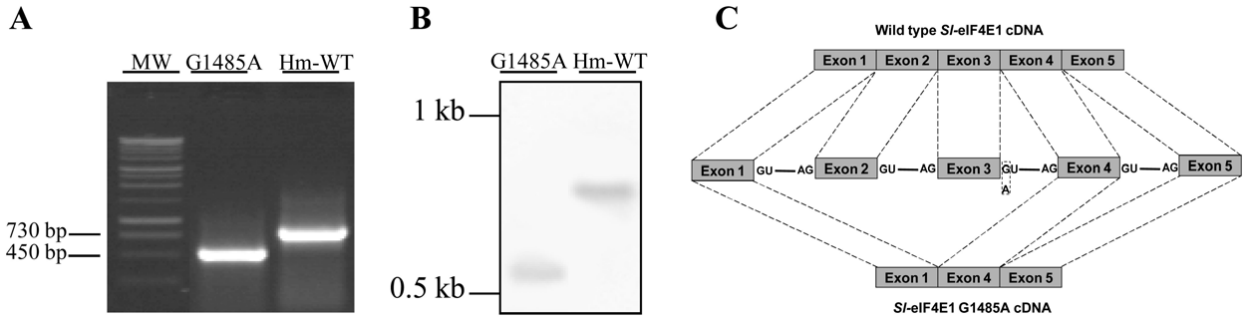
cDNA analysis of *Sl*-eIF4E1 G1485A splicing mutant. (**A**) Total RNA was extracted from leaf tissues of the Hm-WT and G1485A mutant and full lengh cDNAs were amplified and analysed on 1% agarose gel. (**B**) Northern analysis of *Sl*-eIF4E1 from Hm-WT and G1485A mutant lines, using exon 1 as probe. (**C**) Representation of the cDNA structure of the wildtype and the G1485A mutant form. The G1485A mutation is shown by a dashed rectangle.

The G1485A mutation is located in an intron encoding splice site. To test whether this mutation affect the splicing of *Sl-eIF4E1*, we designed primers to amplify the full-length cDNA of the gene. The forward primer was designed on the 5′ UTR of the first exon encompassing the start codon and the reverse primer was the adapter primer anchored on the 3′ end UTR. RT-PCR was performed on cDNA made from RNA of *Sl-eIF4E1* G1485A line and Hm-WT leaves. The amplified Hm-WT cDNA was of the expected size (730 bp) and the sequence fitted with the known exon-intron organization of the plant *Sl-eIF4E* genes [46]. In contrast, the amplified cDNA of the *Sl*-eIF4E1 G1485A allele was significantly smaller than the one from Hm-WT (450 bp), which suggested an alteration in intron splicing (Figure 3A). Sequence analysis of the amplified *Sl*-eIF4E1 G1485A cDNA showed that both the second and the third exons were missing (Figure 3B and 3C). The deletion of exons two and three in the mutant line was further confirmed by northern blot analysis using a *Sl*-*eIF4E1* antisense RNA probe complementary to exon 1 (Figure 3B).

### The *Sl*-eIF4E1 G1485A truncated protein is impaired in cap-binding activity

**Figure 4:**
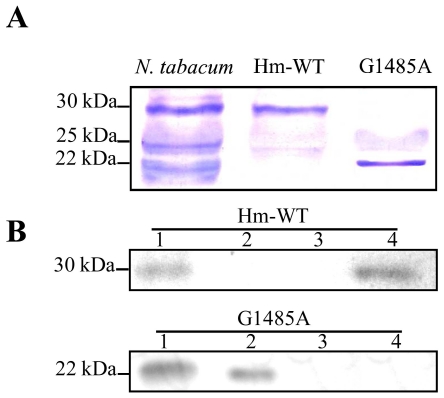
Protein analysis of *Sl*-eIF4E1 G1485A splicing mutant. (**A**) Western blot analysis of total soluble leaf protein of *N. tabacum*, *S. lycopersicum* Hm-WT and *Sl*-eIF4E1 G1485A mutant probed with an antibody raised against *N. tabacum* Nt-eIF4E1. (**B**) Soluble protein extracts of the Hm-WT and G1485A mutant were purified by affinity chromatography on m7G-sepharose column. Total protein extract (lane 1), the flow through (lane 2), the wash (lane 3) and the bound eIF4E proteins eluted with an m7GDP-cap analogue were analysed by Western blot, using Nt-eIF4E antibody.

Total leaf proteins extracted from *Sl*-eIF4E1 G1485A, Hm-WT and *Nicotiana tabacum* cv. Xanthii (control) plants were probed with a polyclonal antibody raised against bacterially expressed *Nt-eIF4E* cDNA in a western blot analysis. This antibody is known to cross-react with tobacco eIF(iso)4E [47]. Three polypeptides were detected from tobacco leaf extracts, migrating at 30, 25 and 22 kDa corresponding to Nt-eIF4E and two isoforms of Nt-eIF(iso)4E, respectively (Figure 4A). In tomato, a single polypeptide of 30 kDa was detected in leaf extract of the Hm-WT and as expected a polypeptide of approximatively 22 kDa corresponding to the mutant form was detected in Sl-eIF4E1 G1485A (Figure 4A).

To address the functional consequence of the G1485A mutation of *Sl*-eIF4E1 for cap-binding activity, soluble protein extracts of the Hm-WT and the G1485A mutant line were purified by affinity chromatography on m7G-sepharose column. After purification, the fractions, the flow through, the wash and the bound forms were subjected to western blot analysis, using Nt-eIF4E antibody (Figure 4B). A single polypeptide of 30 kDa that bound to the m7G-sepharose and is eluted with m7GDP-cap analogue was detected in the Hm-WT, whereas no polypeptide could be detected in the bound fraction of the G1485A mutant (Figure 4B, lane 4). On the other hand, a single polypeptide of 22 kDa was detected in the flow through fraction of the G1485A mutant (Figure 4B, lane 2). All together these results indicate that the *Sl*-eIF4E1 G1485A splicing mutant encode for a truncated eIF4E1 protein that is impaired in cap-binding activity.

## Discussion

Several EMS-mutagenized populations have been described in tomato, however, information on the quality of the mutagenesis and production and maintenance of the seed stocks are often unavailable. We have chosen to set up the TILLING platform on M82 mutant collection for which phenotypic data based on visual characterization of M2 plants from young seedling to fruit maturation stages were generated and categorized into a morphological catalogue that can be searched and accessed via the web ([42]; http://zamir.sgn.cornell.edu/mutants/). M82 is also a cultivar that performs well under various growth environments and many genetic resources were created based on this genetic material.

In order to exploit the mutant population using reverse genetics, genomic DNA was prepared from the mutant lines via high-throughput automated protocols and organized in pools for bulked screening. Although DNA sequence methods are considered the golden standard for mutation discovery, identification of rare mutations in large populations using next-generation sequencing machines is still a challenge. This is mainly due to the high frequency of sequence errors, and thus, identification of true mutants requires statistics methods and validation of large numbers of putative mutations [48], [49], [50], [51]. In this work, we have chosen to till candidate genes using the ENDO1 system, mainly because of its low cost and the low number of false positives [45], [52].

In our TILLING screens of the 19 genes, on average, we identified 8 alleles per tilled kilobase, estimated from the tilled 30.9 kb and the 256 identified alleles. We also calculated the overall mutation rate of one mutation every 574 kb in M82 mutant collection. This mutation frequency is 2-fold lower than the rate of one mutation per 300 kb reported for *A. thaliana* [21] or one mutation per 200 kb reported for *Pisum sativum* [52], and 2-fold higher than the rate of one mutation per megabase reported for barley [53]. A much more saturated mutation density has been observed in polyploid species (1/40 kb in tetraploïd wheat and 1/24 kb in hexaploïd wheat) [54], however such species are able to withstand much higher doses of EMS without obvious impact on survival or fertility rates, probably due to multiple gene redundancies in their polyploid genomes. The systematic analysis of 4 000 induced mutations in *Escherichia coli* lac repressor, reported in [55], has revealed that 41% of the alterations affect the protein function. Thus 8 alleles per kilobase obtained in our TILLING screens would be sufficient to identify mutations that alter the function of the tilled protein.

Translation initiation factor-mediated virus resistance is a wide spread mechanism in plants [32]. To engineer new resistance alleles we screened for mutations in translation factors and identified 83 alleles out of which 7 were in eIF4E1. Because potyvirus resistance in a range of plant species was previously demonstrated to result from amino acid changes or knock-out of eIF4E or eIF(iso)4E proteins, resistance assays were carried out on these mutants. Among the 7 eIF4E1 mutants, resistance to potyvirus was only identified in the Sl-eIF4E1 G1485A mutant line. The feature of this mutant is that it is affected in the pre-mRNA splicing of Sl-eIF4E1 and encodes for a truncated mRNA lacking exons 2 and 3. The putative encoded protein is therefore 106 amino acids long in comparison with the 231 amino acids of the wild type protein. Several amino acids demonstrated to be hallmarks of functional eIF4E proteins are lacking, including amino acids involved in cap-binding and stabilization of the protein structure [56]. The inability of Sl-eIF4E1 G1485A to bind m7G-sepharose column *in vitro* confirmed that this truncated eIF4E1 protein is non-functional for cap-binding. Nevertheless, plants homozygous for the mutation do not display any obvious growth and developmental phenotypes, in agreement with the known ability of eIF4E proteins to compensate for one another in cellular functions [39], [47], [57]. Other eIF4E mutants, including those with a stop codon in exon 1 of Sl-eIF4E2 and a stop codon in exon 2 of Sl-eIF(iso)4E, were fully susceptible to potyviruses. Altogether, these results confirm the central role of eIF4E1 for potyvirus resistance in solanaceous crops [32], [34].

A complete resistance to most of PVY and TEV strains was identified in the wild tomato relatives *S. habrochaites* PI247087. Genetic and functional analyses suggested that this resistance is mediated by a few number of amino acid changes in translation initiation factor eIF4E1 [41]. In link with the result obtained in this study, it is striking that the Sl-eIF4E1 G1485A mutant line is immune to PVY-LYE90 and PepMoV-Texas, but susceptible to other common strains of PVY and TEV, including PVY-LYE84 and TEV-HAT. One possible explanation to the narrow resistance spectrum of the Sl-eIF4E1 G1485A mutant line could be that PVY-LYE90 and PepMoV-Texas specifically require Sl-eIF4E1 as host factor for their infectious cycle whereas PVY-LYE84 and TEV-HAT may use more than one eIF4E protein forms to infect the plant. Such a situation was already described in pepper, where simultaneous mutations in translation initiation factors eIF4E1 and eIF(iso)4E are required to prevent infection by the potyvirus *Pepper veinal mottle* virus [39]. This hypothesis will be addressed by obtaining and phenotyping the Sl-eIF4E1 G1485A and Sl-eIF(iso)4E W105Stop double mutants.

A screen for mutations was also performed in translation initiation factors eIF4G and eIF(iso)4G. Among the 43 *Sl*-eIF4G and 16 *Sl*-eIF(iso)4G point mutants obtained, 20 for eIF4G and 4 for eIF(iso)4G respectively correspond to missense mutations. Among these, 4 *Sl*-eIF4G and 4 *Sl*-eIF(iso)4G mutants showed amino acid changes in the MiF4G domain demonstrated to be involved interaction with *Rice yellow mottle virus* [33]. Nevertheless, none of the mutant lines showed virus resistance. Although mutations in eIF4G factors responsible for potyvirus resistance were never identified in the natural diversity of cultivated species, the requirement of eIF4G for potyvirus infection was demonstrated through susceptibility analysis of *A. thaliana* knocked-out for eIF4G genes [58]. As above, functional redundancy between eIF4G and eIF(iso)4G may be the cause of the full susceptibility of the mutant lines.

### Conclusion

In conclusion, our results on eIF4E show that TILLING is an appropriate technology to engineer new resistance alleles to economically important plant viruses, using as target host factors required for the viral infectious cycle. The new eIF4E1 allele, *Sl*-eIF4E1 G1485A, discovered by TILLING could be used as a new genetic resource for potyvirus resistance in tomato breeding programs. As the EMS mutants are nontransgenic, subsequent generations can be grown under field conditions, without restrictions, for phenotypic analysis and advantageous alleles can be immediately incorporated into a fast-paced molecular breeding program using the characterized induced mutations as markers for selection.

## Methods

### Plant material and growing conditions

The *S. lycopersicum* cv. M82. mutant collection was described previously [42]. Plants were grown in a growth chamber at 25°C day, 19°C night, 50% relative humidity and 12h day length.

### Genomic DNA extraction and pooling

Eighteen tomato leaf discs (diameter 10mm) from six individual plants per M3 family were collected in 96-well plates containing 2 steel beads (4mm) per well, and tissues were ground using a bead mill. Genomic DNA was isolated using the Dneasy 96 Plant Kit (Qiagen, Hilden, Germany). DNAs were quantified on a 0.8% agarose gel using λ DNA (Invitrogen, Carlsbad, USA) as a concentration reference. DNA samples were diluted tenfold and pooled eightfold in a 96-well format. A population of 4759 arrayed DNAs from mutagenized individuals is presently available for screening.

### PCR amplification and mutation detection

The GenBank accession numbers of sequences we produced for TILLING are GQ451830 (*Sl-eIF4E1*), GQ451831 (*Sl-eIF4E2*), GQ451832 (*Sl-eIF(iso)4E*), GQ451834 (*Sl-eIF4G*) and GQ451835 (*Sl-eIF(iso)4G*). PCR amplification is based on nested-PCR. The first PCR amplification is a standard PCR reaction using target-specific primers and 4 ng of tomato genomic DNA. One microlitre of the first PCR served as a template for the second nested PCR amplification, using gene-specific inner primers labelled at the 5′end with infra-red dyes IRD700 and IRD800 (see Table 4, LI-COR®, Lincoln, Nebraska, USA). Mutation detection was carried out as described previously [45]. The identity of the mutations was determined by sequencing.

**Table 4:** Primers used to amplify *Sl-eIF4E1*, *Sl-eIF4E2*, *Sl-eIF(iso)4E*, *Sl-eIF4G* and *Sl-eIF(iso)4G* genes and the size of the tilled amplicons.

Gene	Primers name*	Sequence (5′ to 3′)	Fragment size (bp)
SleIF4E1	SleIF4E1-ext-F1	ATGGCAGCAGCTGAAATGGAGAGAACGATGT	395
	SleIF4E1-ext-R1	GACTATCTAAACTTTCTCGAGGATTTG	
	SleIF4E1-F1	TGAAATGGAGAGAACGATGT	331
	SleIF4E1-R1	GTTTTCCCTTACACTACACTATCAC	
	SleIF4E1-ext-F2	GTACAACTTGAACGATGACATACCTG	1381
	SleIF4E1-ext-R3	CTAGTACCTACCAACCTTTCCAGTACG	
	SleIF4E1-F2	TAGGTCTTTGAAATGCTATTATCCT	822
	SleIF4E1-R2	ACGAGACAAAACAATATCACACTTT	
	SleIF4E1-F3	AAGTCATCAGATATATAGGAAGTGC	665
	SleIF4E1-R3	GTGTTTCTTGCAATCCCACACTGCATC	
SleIF4E2	SleIF4E2-ext-F1	GCAGGCGGGACGAAAACACCAAAAATG	471
	SleIF4E2-ext-R1	AGTACTAGAGATTTCTGCTACATGC	
	SleIF4E2-F1	GGGACGAAAACACCAAAAATG	324
	SleIF4E2-R1	AAAACATTAGAAACCCTAATCCTAC	
SleIF(iso)4E	SleIF(iso)4E-ext-F1	CTATACAAAAGGAGAGGATTTTGAC	1381
	SleIF(iso)4E-ext-R1	CAGAGATAGTGGAACAATAACACAG	
	SleIF(iso)4E-F1	GGAGAGGATTTTGACGGGAAAACA	1340
	SleIF(iso)4E-R1	GTTCTGAAGCTATATAACACCAGAG	
SleIF4G	SleIF4G-ext-F1	CTGAACCTTTGGATTCCAGAAATCAGG	1148
	SleIF4G-ext-R1	CCAGGCATCTTATTCCACTTGTCTC	
	SleIF4G-F1	CTTTGGATTCCAGAAATCAGGATGCG	1137
	SleIF4G-R1	CATCTTATTCCACTTGTCTCCATCC	
	SleIF4G-ext-F2	AATACTATCTGGACCAATGCAATCC	979
	SleIF4G-ext-R2	ACTAGGAGTACGAGCAAGCCTAGTAGC	
	SleIF4G-F2	CACAGAATATGCACAAAGCTGAAGT	846
	SleIF4G-R2	TAGGAGTACGAGCAAGCCTAGTAGC	
	SleIF4G-ext-F3	ATACATGCCTGAGCGATTATCTAGT	1698
	SleIF4G-ext-R3	CCCCCACCTATTAAAACCCTAAATA	
	SleIF4G-F3	GCCTGAGCGATTATCTAGTCAACAT	1626
	SleIF4G-R3	AAAGTTCTCAAGCCGCATAGTAGAG	
SleIF(iso)4G	SleIF(iso)4G-ext-F1	CTCTCTCTGTATCCCTTTCCATCTCT	1709
	SleIF(iso)4G-ext-R1	TTTAGTACATCAGGTGTCAAACTTGC	
	SleIF(iso)4G-F1	GTTTTCACTTGCACATGAGACTACTAC	1300
	SleIF(iso)4G-R1	GATAATGACAACTGGGAAGTTCCTA	
	SleIF(iso)4G-ext-F2	AACTAGGAAGATGCCTGGTACACCT	1156
	SleIF(iso)4G-ext-R2	CAAAGAGAGGAGATAAACAAATAACT	
	SleIF(iso)4G-F2	ACACCTGATGTACTAAAGAGAAAAA	717
	SleIF(iso)4G-R2	AAATCTTTCTTTCAACATACTACTACC	

*F and R indicate forward and reverse primers, respectively.

### Potyvirus strains and disease resistance evaluation

All plants were grown under greenhouse conditions and transferred into growth chambers before inoculation. The susceptibility of M82, homozygous mutant lines and their corresponding homozygous wild type (Hm-WT) to PVY, PepMoV and TEV were determined by mechanical inoculation of 12 plants per genotype at the two-leaf stage using PVY-LYE84 [59], PVY-LYE90 [59], TEV-HAT [60] and PepMoV-Texas strains [61]. PVY and PepMoV strains were maintained on *Capsicum annuum* Yolo Wonder plants and TEV strain on *Datura stramonium* plants, respectively, and transferred every 4–8 weeks. Inoculum and mechanical inoculation procedures were as described previously [62]. Fifteen days post-inoculation (dpi), systemic infection was assayed by determining the presence/absence of symptoms on non-inoculated leaves and confirmed by DAS-ELISA using PVY, TEV or PepMoV antibodies.

PVY and PepMoV RNAs accumulation were assessed by RT-PCR on inoculated and upper non-inoculated leaves of the *Sl*-eIF4E1 splicing mutant G1485A, the corresponding Hm-WT and M82. Total RNA was isolated from leaf tissue using TRI Reagent (Sigma, Aldrich) and 2 µl was used for reverse transcription followed by polymerase chain reaction (RT-PCR). RT-PCR for PVY and PepMoV were performed with primers specific for the VPg (PVY-forward, 5′-GGCAAGAATAAATCCAAGAGAATA-3′; PVY-reverse, 5′- TTCATGCTCTACTTCTTGACTGGG-3′; PepMoV-forward, 5′-AGAGGATCCTAGGACGCTCTAAGACGAAAAGAATT-3′; PepMoV-reverse, 5′-ATAGTCGACTTTATTCGTGCTTCACAACTTCCTTTGG-3′). RT-PCR for *Sl-eIF4E1* cDNA amplification was used as control.

### 3′-RACE PCR and northern analysis

Total RNA was isolated from 100 mg leaf tissues of the *Sl*-eIF4E1 G1485A splicing mutant and its corresponding Hm-WT using Tri-Reagent (Sigma-Aldrich, St Louis, USA). 3′ RACE-PCR was performed using the GIBCO/BRL Life Technologies 3′ RACE System. In a first step, a nested 3′ RACE-PCR was performed with a forward primer encompassing the *Sl*-eIF4E1 start codon (5′-ATGGCAGCAGCTGAAATGGAGAGA-3′) in combination with the adapter primer (AUAP) of the kit. A dilution (1/100) of this PCR was used for the second PCR with a forward primer hybridizing in exon 1 of *Sl*-eIF4E1 (5′-GCATCGTATTTAGGGAAAGAAATC-3′) and the AUAP primer. All amplifications were performed with High Fidelity Platinium Taq polymerase (GIBCO/BRL, Life Technologies). PCR products were sequenced by Genome Express (Grenoble, France). Northern blot analysis was performed using standard procedures [63] using a [^32^P]-labelled *Sl*-eIF4E1 antisense RNA probe complementary to exon 1 of the *Sl*-eIF4E1 cDNA.

### Protein purification and cap-affinity chromatography

Total proteins were extracted by grinding 100 mg of leaf tissues in Laemmli buffer (0.125 mM Tris-HCl pH6.8, 10% β-mercaptoethanol, 4% SDS, 0.004 mM Bromophenol blue and 20% glycerol). For cap-binding analysis, total soluble proteins were extracted by grinding 500 mg of leaf tissues in 1 ml of extraction buffer (20 mM HEPES pH 7.6, 0.1 M KCl, 2 mM EDTA and 5% Glycerol). After centrifugation (15 600 g, 5 min), the supernatant was recovered and incubated with 100 µl of m^7^GTP Sepharose 4B (Amersham Biotech) at 4°C for 16h. The beads were pelleted for 2 min at 15 600g and washed extensively with extraction buffer. The proteins retained were eluted with extraction buffer containing 100 µM m^7^GDP. The different fractions (elution, wash and supernatant) were analyzed by western blotting. Proteins were resolved using standard 12.5% sodium dodecyl sulphate-polyacrylamide gel electrophoresis and transferred to a 0.22 µm-pore-size nitrocellulose membrane by electroblotting. The membranes were blocked with 3% BSA-TTBS (20 mM Tris pH 7.5, 150 mM NaCl, 0.1% Tween) and were incubated with the Nt-eIF4E antibody (gift from David Twell, Univ. Leicester, UK, [47] diluted 1/1 000 with 3% BSA-TTBS for 1 h at room temperature. Then, membranes were washed 3 times in TTBS buffer and incubated with alkaline phosphatase–conjugated anti-rabbit antibodies diluted 1/10000 for 1 hour. After washing, the signal was visualized with nitroblue tetrazolium.

## References

[1] Pedley KF, Martin GB (2003). Molecular basis of Pto-mediated resistance to bacterial speck disease in tomato.. Annu Rev Phytopathol.

[2] Gillaspy G, Ben-David H, Gruissem W (1993). Fruits: A Developmental Perspective.. Plant Cell.

[3] Alba R, Payton P, Fei Z, McQuinn R, Debbie P (2005). Transcriptome and selected metabolite analyses reveal multiple points of ethylene control during tomato fruit development.. Plant Cell.

[4] Giovannoni J (2001). Molecular Biology of Fruit Maturation and Ripening.. Annu Rev Plant Physiol Plant Mol Biol.

[5] Giovannoni JJ (2007). Fruit ripening mutants yield insights into ripening control.. Curr Opin Plant Biol.

[6] Wilkinson JQ, Lanahan MB, Yen HC, Giovannoni JJ, Klee HJ (1995). An ethylene-inducible component of signal transduction encoded by never-ripe.. Science.

[7] Carrari F, Baxter C, Usadel B, Urbanczyk-Wochniak E, Zanor MI (2006). Integrated analysis of metabolite and transcript levels reveals the metabolic shifts that underlie tomato fruit development and highlight regulatory aspects of metabolic network behavior.. Plant Physiol.

[8] Ohyama A, Ito H, Sato T, Nishimura S, Imai T (1995). Suppression of acid invertase activity by antisense RNA modifies the sugar composition of tomato fruit.. Plant Cell Physiol.

[9] Robinson NL, Hewitt JD, Bennett AB (1988). Sink Metabolism in Tomato Fruit: I. Developmental Changes in Carbohydrate Metabolizing Enzymes.. Plant Physiol.

[10] Bramley PM (2002). Regulation of carotenoid formation during tomato fruit ripening and development.. J Exp Bot.

[11] Isaacson T, Ronen G, Zamir D, Hirschberg J (2002). Cloning of tangerine from tomato reveals a carotenoid isomerase essential for the production of beta-carotene and xanthophylls in plants.. Plant Cell.

[12] Frary A, Nesbitt TC, Grandillo S, Knaap E, Cong B (2000). fw2.2: a quantitative trait locus key to the evolution of tomato fruit size.. Science.

[13] Wang Y, Li J (2008). Molecular basis of plant architecture.. Annu Rev Plant Biol.

[14] Doganlar S, Frary A, Ku HM, Tanksley SD (2002). Mapping quantitative trait loci in inbred backcross lines of Lycopersicon pimpinellifolium (LA1589).. Genome.

[15] D'Agostino N, Aversano M, Frusciante L, Chiusano ML (2007). TomatEST database: in silico exploitation of EST data to explore expression patterns in tomato species.. Nucleic Acids Res.

[16] Fei Z, Tang X, Alba RM, White JA, Ronning CM (2004). Comprehensive EST analysis of tomato and comparative genomics of fruit ripening.. The Plant Journal.

[17] Moco S, Bino RJ, Vorst O, Verhoeven HA, de Groot J (2006). A Liquid Chromatography-Mass Spectrometry-Based Metabolome Database for Tomato.. Plant Physiol.

[18] Stack SM, Royer SM, Shearer LA, Chang SB, Giovannoni JJ (2009). Role of fluorescence in situ hybridization in sequencing the tomato genome.. Cytogenet Genome Res.

[19] Alonso JM, Stepanova AN, Leisse TJ, Kim CJ, Chen H (2003). Genome-wide insertional mutagenesis of Arabidopsis thaliana.. Science.

[20] Waterhouse PM, Graham MW, Wang MB (1998). Virus resistance and gene silencing in plants can be induced by simultaneous expression of sense and antisense RNA.. Proc Natl Acad Sci U S A.

[21] Greene EA, Codomo CA, Taylor NE, Henikoff JG, Till BJ (2003). Spectrum of Chemically Induced Mutations From a Large-Scale Reverse-Genetic Screen in Arabidopsis.. Genetics.

[22] Comai L, Henikoff S (2006). TILLING: practical single-nucleotide mutation discovery.. Plant J.

[23] Henikoff S, Till BJ, Comai L (2004). TILLING. Traditional mutagenesis meets functional genomics.. Plant Physiol.

[24] McCallum CM, Comai L, Greene EA, Henikoff S (2000). Targeting induced local lesions IN genomes (TILLING) for plant functional genomics.. Plant Physiol.

[25] Bentley A, MacLennan B, Calvo J, Dearolf CR (2000). Targeted recovery of mutations in Drosophila.. Genetics.

[26] Coghill EL, Hugill A, Parkinson N, Davison C, Glenister P (2002). A gene-driven approach to the identification of ENU mutants in the mouse.. Nat Genet.

[27] Colbert T, Till BJ, Tompa R, Reynolds S, Steine MN (2001). High-throughput screening for induced point mutations.. Plant Physiol.

[28] Perry JA, Wang TL, Welham TJ, Gardner S, Pike JM (2003). A TILLING reverse genetics tool and a web-accessible collection of mutants of the legume Lotus japonicus.. Plant Physiol.

[29] Leonard S, Plante D, Wittmann S, Daigneault N, Fortin MG (2000). Complex formation between potyvirus VPg and translation eukaryotic initiation factor 4E correlates with virus infectivity.. J Virol.

[30] Revers Fdr, Le Gall O, Candresse T, Maule AJ (1999). New Advances in Understanding the Molecular Biology of Plant/Potyvirus Interactions.. Molecular Plant-Microbe Interactions.

[31] Maule A, Caranta C, Boulton M (2007). Sources of natural resistance to plant viruses: status and prospects.. Molecular Plant Pathology.

[32] Robaglia C, Caranta C (2006). Translation initiation factors: a weak link in plant RNA virus infection.. Trends Plant Sci.

[33] Albar L, Bangratz-Reyser M, Hebrard E, Ndjiondjop MN, Jones M (2006). Mutations in the eIF(iso)4G translation initiation factor confer high resistance of rice to Rice yellow mottle virus.. Plant J.

[34] Charron C, Nicolai M, Gallois JL, Robaglia C, Moury B (2008). Natural variation and functional analyses provide evidence for co-evolution between plant eIF4E and potyviral VPg.. Plant J.

[35] Kang BC, Yeam I, Frantz JD, Murphy JF, Jahn MM (2005). The pvr1 locus in Capsicum encodes a translation initiation factor eIF4E that interacts with Tobacco etch virus VPg.. Plant J.

[36] Yeam I, Cavatorta JR, Ripoll DR, Kang BC, Jahn MM (2007). Functional dissection of naturally occurring amino acid substitutions in eIF4E that confers recessive potyvirus resistance in plants.. Plant Cell.

[37] Gallie DR, Browning KS (2001). eIF4G functionally differs from eIFiso4G in promoting internal initiation, cap-independent translation, and translation of structured mRNAs.. J Biol Chem.

[38] Hwang J, Li J, Liu WY, An SJ, Cho H (2009). Double mutations in eIF4E and eIFiso4E confer recessive resistance to Chilli veinal mottle virus in pepper.. Mol Cells.

[39] Ruffel S, Gallois JL, Moury B, Robaglia C, Palloix A (2006). Simultaneous mutations in translation initiation factors eIF4E and eIF(iso)4E are required to prevent pepper veinal mottle virus infection of pepper.. J Gen Virol.

[40] Parrella G, Ruffel S, Moretti A, Morel C, Palloix A (2002). Recessive resistance genes against potyviruses are localized in colinear genomic regions of the tomato (Lycopersicon spp.) and pepper (Capsicum spp.) genomes.. Theor Appl Genet.

[41] Ruffel S, Gallois JL, Lesage ML, Caranta C (2005). The recessive potyvirus resistance gene pot-1 is the tomato orthologue of the pepper pvr2-eIF4E gene.. Mol Genet Genomics.

[42] Menda N, Semel Y, Peled D, Eshed Y, Zamir D (2004). In silico screening of a saturated mutation library of tomato.. Plant J.

[43] Till BJ, Reynolds SH, Greene EA, Codomo CA, Enns LC (2003). Large-scale discovery of induced point mutations with high-throughput TILLING.. Genome Res.

[44] Rozen S, Skaletsky H (2000). Primer3 on the WWW for general users and for biologist programmers.. Methods Mol Biol.

[45] Triques K, Sturbois B, Gallais S, Dalmais M, Chauvin S (2007). Characterization of Arabidopsis thaliana mismatch specific endonucleases: application to mutation discovery by TILLING in pea.. Plant J.

[46] Ruffel S, Caranta C, Palloix A, Lefebvre V, Caboche M (2004). Structural analysis of the eukaryotic initiation factor 4E gene controlling potyvirus resistance in pepper: exploitation of a BAC library.. Gene.

[47] Combe JP, Petracek ME, van Eldik G, Meulewaeter F, Twell D (2005). Translation initiation factors eIF4E and eIFiso4E are required for polysome formation and regulate plant growth in tobacco.. Plant Mol Biol.

[48] Hoffmann C, Minkah N, Leipzig J, Wang G, Arens MQ (2007). DNA bar coding and pyrosequencing to identify rare HIV drug resistance mutations.. Nucleic Acids Res.

[49] Huse SM, Huber JA, Morrison HG, Sogin ML, Welch DM (2007). Accuracy and quality of massively parallel DNA pyrosequencing.. Genome Biol.

[50] Rigola D, van Oeveren J, Janssen A, Bonne A, Schneiders H (2009). High-throughput detection of induced mutations and natural variation using KeyPoint technology.. PLoS One.

[51] Wang C, Mitsuya Y, Gharizadeh B, Ronaghi M, Shafer RW (2007). Characterization of mutation spectra with ultra-deep pyrosequencing: application to HIV-1 drug resistance.. Genome Res.

[52] Dalmais M, Schmidt J, Le Signor C, Moussy F, Burstin J (2008). UTILLdb, a Pisum sativum in silico forward and reverse genetics tool.. Genome Biology.

[53] Caldwell DG, McCallum N, Shaw P, Muehlbauer GJ, Marshall DF (2004). A structured mutant population for forward and reverse genetics in Barley.. The Plant Journal.

[54] Slade AJ, Fuerstenberg SI, Loeffler D, Steine MN, Facciotti D (2005). A reverse genetic, nontransgenic approach to wheat crop improvement by TILLING..

[55] Markiewicz P, Kleina LG, Cruz C, Ehret S, Miller JH (1994). Genetic Studies of the lac Repressor. XIV. Analysis of 4000 Altered Escherichia coli lac Repressors Reveals Essential and Non-essential Residues, as well as “Spacers” which do not Require a Specific Sequence.. Journal of Molecular Biology.

[56] Marcotrigiano J, Gingras AC, Sonenberg N, Burley SK (1997). Cocrystal structure of the messenger RNA 5′ cap-binding protein (eIF4E) bound to 7-methyl-GDP.. Cell.

[57] Duprat A, Caranta C, Revers F, Menand B, Browning KS (2002). The Arabidopsis eukaryotic initiation factor (iso)4E is dispensable for plant growth but required for susceptibility to potyviruses.. Plant J.

[58] Nicaise V, Gallois JL, Chafiai F, Allen LM, Schurdi-Levraud V (2007). Coordinated and selective recruitment of eIF4E and eIF4G factors for potyvirus infection in Arabidopsis thaliana.. FEBS Lett.

[59] Moury B, Morel C, Johansen E, Guilbaud L, Souche S (2004). Mutations in Potato virus Y Genome-Linked Protein Determine Virulence Toward Recessive Resistances in Capsicum annuum and Lycopersicon hirsutum.. Molecular Plant-Microbe Interactions.

[60] Schaad MC, Anderberg RJ, Carrington JC (2000). Strain-Specific Interaction of the Tobacco Etch Virus NIa Protein with the Translation Initiation Factor eIF4E in the Yeast Two-Hybrid System.. Virology.

[61] Vance V, Moore D, Turpen T, Bracker A, Hollowell V (1992). The complete nucleotide sequence of pepper mottle virus genomic RNA: comparison of the encoded polyprotein with those of other sequenced potyviruses.. Virology.

[62] Caranta C, Palloix A (1996). Both common and specific genetic factors are involved in polygenic resistance of pepper to several potyviruses.. Theoretical and Applied Genetics.

[63] Sambrook J, Frisch E, Maniatis T (1989). Molecular cloning: a laboratory manual. 2nd eds.

